# Negative Pressure Wound Therapy with Instillation in the Septic Open Abdomen Utilizing a Modified Negative Pressure Therapy System

**DOI:** 10.1016/j.amsu.2018.10.007

**Published:** 2018-10-10

**Authors:** Pablo Sibaja Alvarez, Alfredo Sánchez Betancourt, Luis G. Fernández

**Affiliations:** a Universidad Federada San Judas Tadeo, San Jose, Costa Rica; b Christus Trinity Mother Frances Health System, Tyler, Texas, USA

**Keywords:** Open abdomen. Sepsis, Negative pressure wound therapy, instillation therapy, NPWT-i

## Abstract

**Background:**

Various treatment modalities are utilized to treat the open abdomen. The use of negative pressure wound therapy(NPWT)has been a great advancement and has become the preferred modality for temporary abdominal closure technique (TAC). Programmed instillation of the abdominal cavity with saline solution in conjunction with a commercial negative pressure system showed positive results in the management of severe abdominal sepsis in patients that were treated with an open abdomen. Severe abdominal sepsis continues to be an oftendifficult clinical problem for the general surgeon. The use of an open abdomen technique in this setting and the ideal TAC method continue to be debated. The failure to understand the biomechanical features/limitations of negative pressure devices are often contributing factors associated with therapeutic failures reported in the literature.

**Objectives:**

To describe the underlying principles behind negative pressure wound therapy with instillation in the context of abdominal sepsis, as well as its optimal usage in these conditions.

**Methods:**

A systematic review and two retrospective cohort studies, both published and unpublished performed by some of the authors were included to provide a basis form comparison between NPWT and NPWT-I outcomes in managing abdominal sepsis.

**Conclusion:**

Our findings suggest that this technique appears to reduce morbidity, mortality, and hospital and critical care length of stay. This communication is intended to help inform general surgeons that manage complex abdominal infections on how to optimally apply this technique.

## Highlights

• Instillation of saline solution into the septic open abdomen with the use of a modified Negative Pressure Device is a novel approach to management of abdominal sepsis.
• This Temporary Abdominal Closure method was previously described as a viable alternative to traditional NPWT.
• Many considerations must be taken into account when applying this method such as length and width of the foam dressing, the size of the ostomy wound, the pressure applied by the NPWT machine, the soak or dwell time provided for the instilled fluid and the periodicity of the dressing changes.

## Background

**Figure 1:**
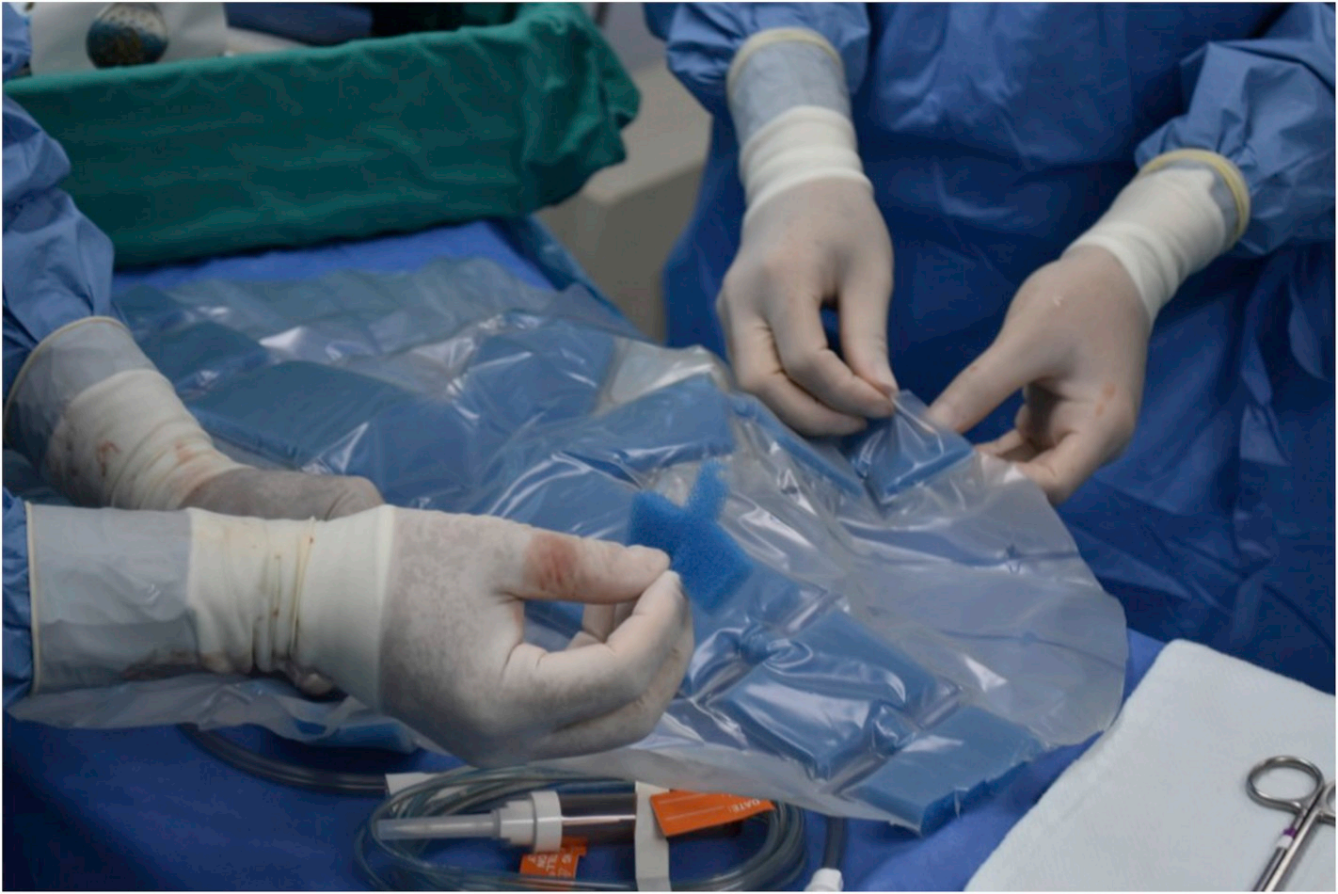

**Figure 2:**
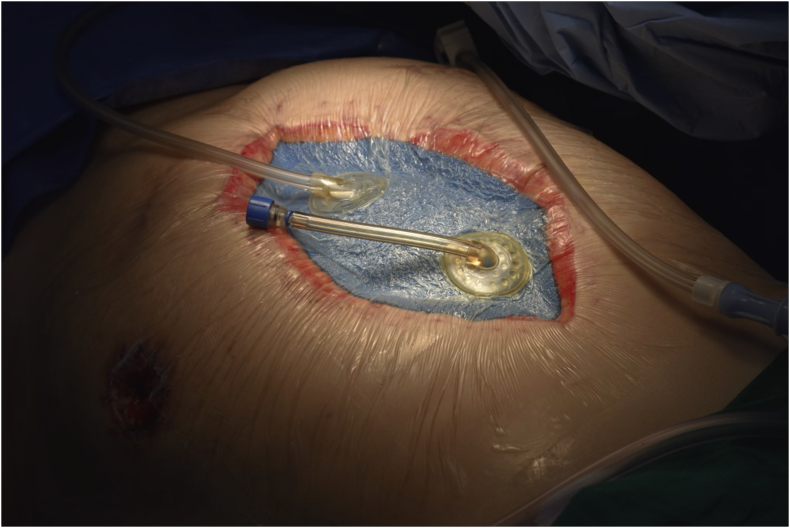

The open abdomen (OA) technique has been shown to be a beneficial tool in patients with complex abdominal injury and sepsis. This form of surgical intervention (also known as laparostomy) was initially used as a last resort in cases where there was an inability to close the abdominal wall due to tissue loss or extreme visceral edema. The use of the OA in the treatment of abdominal compartment syndrome (ACS) and as a component of damage control strategy in trauma patients (1) have increased. (see Figs. 1 and 2)

Temporary abdominal closure (TAC) in OA patients was initially carried out using only “*passive”* methods, which provided coverage of the abdominal contents and facilitated revisions in the operating room (2).The rationale of maintaining an open abdominal cavity has remained the same; it allows for a step-by-step approach, facilitating patient recovery by permitting the surgeon to closely monitor and opportunely intervene in severe intra-abdominal pathology and its associated complications. Novel variants have arisen, such as evaluations through the use of visceral oximetry (3), prompting early detection of intestinal tissue ischemia, especially in patients with hypovolemia, severe sepsis or systemic inflammatory response syndrome (4).

There are three common indications for leaving the abdominal cavity open: physiological, anatomical or logistical criteria (5), where the physiological variety (eg, low pH, high lactate, and hypotension) is the most common criteria present in patients that require management with this type of approach.

There are multiple techniques associated with the management of the OA, as to facilitate a TAC. They range from loose packing of the abdominal cavity (6), usage of towel clips (7), placement of mesh material (8), polyvinyl bags (9) or even textile and zipper like devices (10). More recently, the use of negative pressure therapy, deployed in a programmed fashion, has been utilized in this context (11,12). Until 2016 (13), there was no consensus as to which treatment option was superior, although various studies indicated that negative pressure wound therapy (NPWT) and its variants were the most effective approach (13), yielding the best results while reducing associated complications. While every method has benefits and downfalls, some options appear to be associated with a higher rate of sequelae. These complications have proven to be of great importance, constituting an equal or higher risk of mortality to patients as the underling pathology that prompted the application of an OA approach. Most complications have been related with repeated and protracted visceral exposure and manipulation; therefore, the use of simpler techniques of TAC utilizing silo closure methods or Bogota bag (Borraez Bag) has been slowly substituted by methods that require less surgical revision, such as NPWT.

A variant of this technique, in which negative pressure was applied concomitantly with instillation of saline solution, was successfully applied to chronic infected soft tissue wounds (14), mainly of the upper and lower extremities (15). Its use in catastrophic abdominal pathology, especially severe intra-abdominal sepsis (16), both by bacteria and fungi (17), has been successfully demonstrated; therefore, it is increasingly considered as an appropriate choice for managing the OA.

The principals involved in the therapeutic effects of NPWT plus instillation (NPWT-i) and the new treatment modalities, such as the newer commercial abdominal negative pressure systems and its proper application in the abdominal cavity will be further discussed in the article.

## Methods

The main objective of our article is to provide the reader with a systematic approach that will allow them to apply NPWT-i in patients with severe abdominal sepsis, as well as the underlying principles that make this therapy a viable OA approach. A literature review was performed utilizing Pubmed, Ebscohost and GoogleScholar using the following terms: negative pressure wound therapy with instillation, septic open abdomen, abdominal instillation and negative pressure wound therapy. Articles relating to the principles and usage of NPWT-I were included. PRISMA criteria were followed in the draft and review of the article (18). The articles included were dated between 1990 and 2018. Articles dealing in NPWT-I and conventional NPWT for management of conditions other than abdominal sepsis were not included unless pertaining to the underlying dynamics of negative pressure therapy. Articles in Spanish and English were included. Due to the small amount of literature relating to this subject, there was no systematic exclusion of studies based on length of follow-up, patient number, instillation volume used, type of instilled fluid or other considerations. No meta-analysis regarding NPWT-I were identified. All studies, except for one performed by the authors were published in scientific journals. Additionally, two retrospective cohort studies, both published and unpublished performed by some of the authors were included to provide a basis for comparison between NPWT and NPWT-I outcomes in managing abdominal sepsis.

### Overview

NPWT was originally postulated in the treatment of peripheral chronic wounds (19), and over time, gained acceptance as a valid treatment option for other conditions. Its use in complex abdominal pathology, in conjunction with an OA, has been widely applied, especially in the context of ACS. Several clinical studies have shown that the basis of these positive results are related to a better control of infectious source, reduction of edema, improved microcirculation and maintenance of a favorable abdominal environment, allowing the body to heal in a shorter timeframe. It is theorized that NPWT is superior in the reduction of cytokines in the abdominal fluid (11), as well as in the blood, although some evidence shows that this is not necessarily correct (20). This reduction would account for the attenuated inflammatory systemic response in patients receiving this therapy. In regards to the effect on bacterial loads, animal models have shown important reductions in the amount of bacteria in wounds (19), which appears to affect certain microorganisms more than others (21), although there are some conflicting reports regarding these results. Due to the complexity of the studies, the actual rate of bacterial bioburden reduction remains unclear (22), and some studies have postulated that there is no influence on bacterial survival with NPWT.

Improvements on wound microcirculation and tissue granulation have been shown in both animal and human models with NPWT. This therapy increases capillary volume and blood flow and volume to applied areas, as well as promoting neovascularization and basement membrane integrity (23). Larger volumes of tissue granulation are obtained due to the increase in necrotic tissue debridement that is caused by the negative pressure, both at high and low pressure settings (24).

The use of a damage control strategy (25) in the context of abdominal sepsis has allowed for a controlled and systematic approach to dealing with the infectious foci, as well as the inflammatory response, positively impacting morbidity and mortality.

The introduction of a two-way therapy (ie, NPWT-i) takes the potential positive effects of traditional negative pressure approach a step further. The essential change is that NPWT-i shifts from a single step therapy to a three-phase cycle: aspiration, instillation and soak time. The inclusion of these additional phases contributes to better control of contaminated fluid residue produced or trapped within the abdominal cavity. The purpose of providing a controlled saline instillation to the abdominal cavity is to mix the instilled solution with the contents, thus dissolving material that would not normally circulate because of high viscosity or deep location within the cavity. The instilled fluid serves as a facilitator for evacuation via negative pressure aspiration. The soak time allows for a more homogeneous mixture. During the aspiration or NPWT phase, the diluted fluids will easily flow towards the aspiration port and leave the abdominal cavity into a sealed container, thus eliminating the threat of septic material dissemination. This material has been shown to be responsible for the inflammatory response syndrome, as demonstrated by the Kubiak´s animal model experiment (11). This approach is clearly focused towards the treatment of a contaminated or septic wound or cavity.

### Classification of the Open Abdomen

In order to better asses and stratify the severity of the condition requiring management with an OA, there have been many different classifications proposed, some relating to the condition of the wound (26) and others relating to the condition of the abdomen and its contents (27). The latter has been the most widely used and accepted classification. The Björk classification (28) (Table 1) was modified in 2016 to give a higher level of severity to the frozen abdomen in conjunction with a potential complication of NPWT, the enteroatmospheric fistulae.

**Table 1:** Amended Björk Classification of the open abdomen.

1A	Clean, no fixation
2A	Contaminated, no fixation
3A	Enteric leak, no fixation
2A	Clean, developing fixation
2B	Contaminated, developing fixation
2C	Enteric leak, developing fixation
3A	Clean, frozen abdomen
3B	Contaminated, frozen abdomen
4	Established enteroatmospheric fistula, frozen abdomen

Source: Björck M, Kirkpatrick AW, Cheatham M, Kaplan M, Leppäniemi A, de Waele JJ. Amended classification of the open abdomen. Scand J Surg. 2016;105 (1).

### Negative Pressure Wound Therapy with Instillation

This treatment modality was first employed in the treatment of infected soft tissue wounds (14, 15) and has been applied in various types of infected wounds in the extremities (29) and to salvage acutely or chronically infected orthopeadic implants (30).

Animal models have shown the benefit of NPWT and NPWT-i in regards to wound granulation (24), but there appears to be a larger reduction of wound size, a reduction of wound exudate and infectious material (31) when using NPWT-i. A reduction in colony forming units and bacterial biofilm also seems to play a role in the mechanism of action of NPWT-i (32).

There are various types of NPWT-i in regards to the frecuency of instillation (31), solutions instilled (29,33) and duration of treatment. With regards to the instillation solution, the selection of chemical compound utilized is associated with the therapeutic goal set by the clinician. Anesthetics can be utilized to provide pain relief (34), antiseptics as well as antibiotics can be utilized for treatment of an infected wound, cavity or implant (35,36). The most commonly instilled solution is 0.9% saline which appears to be as efficient as antimicrobial solutions in the manegement of infected wounds and cavities.

There are few describe contraindications of NPWT-i, one identified refered to the possible association between instillation of the abdominal cavity and hypothermia (28), although, this was not noted in any of the patients treated with NPWT-i by the authors (37).

### NPWT versus NPWT-i for abdominal sepsis the Surgical Intensive Care Unit (SICU) in Hospital Mexico, Costa Rica

Two retrospective cohort studies were performed in the SICU in a public tertiary hospital located in the costa rican capital of San Jose during a five-year period. The first study, which was not published, analyzed the outcomes of 36 patients diagnosed with severe abdominal sepsis that was managed with an open abdomen and NPWT.

The cohort consisted of 20 (56%) males and 16 (44%) females who were managed with an open abdomen and conventional NPWT for abdominal sepsis. The inclusion criteria was having abdominal sepsis, having an APACHE II score of 12 or greater and being managed with an open abdomen and NPWT. The following criteria were documented: overall mortality, hernia rate, fascia closure rate, days till fascia closure was performed, fistula formation and SICU length of stay (LOS). The underlying cause of abdominal contamination was not included in the study.

The outcomes were as follows: 13.8% mortality, 14% hernia rate, 86% fascia closure rate, 14 day SICU LOS. No enteric fistula was detected.

The second cohort study, published in 2017(15), informed of the outcomes of 48 patients managed in the same SICU with an open abdomen and NPWT-i. The cohort was comprised of 20 males and 28 females with an average age of 48 years. The inclusion criteria were as follows: having an APACHE II score of 12 or greater and a Björck open abdomen classification of 2a or greater (2b in the previous classification). Mortality in the NPWT-i group was 8.3%, fascia closure was obtained in 96% patients with a 4% ventral hernia rate, average SICU LOS was 7.4 days. Like in the NPWT group, no enteric fistula was detected. The comparision between the groups are summarized in table 2.

**Table 2:** Comparison of outcomes between Negative Pressure Wound Therapy and Negative Pressure Wound Therapy with instillation.

	NPWT cohort	NPWT-i cohort
Mortality	13.80%	8.30%
Fascia closure rate	86%	96%
Ventral hernia	14%	4%
Enteric fistula	0%	0%
SICU LOS	14 days	7.4 days

### Pitfalls and Limitations of NPWT-I for OA Management

For this treatment to be successful, it is necessary for it to be delivered in the correct way; therefore, a list of actions to be considered during, before and after the initiation of NPWT are as follows.

This technique of OA management has not been adapted to the pediatric patient; therefore, it should be limited to adults. It should be performed only by surgeons who are familiar with the OA approach and understand the risks it entails.

Regarding abdominal wall preparation, care should be taken to thoroughly remove all natural skin oil residues, other oils or Vaseline, adhesive remains, hair and suture material to provide for an adequate adhesive surface. To enhance this step, alcoholic skin cleaners or adhesive enhancers, such as benzoin tincture, can be applied.

The NPWT device acts as a surgical drain; therefore, no additional drains need to be placed.

The greater omentum should not come in contact with the NPWT dressing. This structure, which was commonly used as a protective cover over the visceral mass in previous passive techniques, constitutes a barrier for fluid exchange when using NPWT-i, so it must be properly folded in the upper position to avoid interference.

The presence of a neighboring ostomy does not represent a threat to the integrity of the abdominal seal if the area is properly covered by an adhesive layer and then cut out and covered by a transparent open ostomy bag. Failure of the abdominal seal usually occurs when the surgeon intends to place the adherent layer around the wound opening instead of covering it.

It is important to note that size does matter. A common mistake that hinders the process of installing NPWT is a short and narrow wound with a small abdominal dressing opening. Wound length should be over 15 cm and wound width larger than 10 cm.

Abdominal NPWT-i dressings should be changed every 72 hours, at the most, to prevent material contamination, suction failure or system clogging.

Dressing changes have to be performed within a proper timeframe. For NWPT-i, the dressing changes should be scheduled for periods no longer than 72 hours to prevent dressing soiling and dysfunction. Shorter periods can be considered if a non-functioning or leaking device is detected. A simple way to detect if the foam is saturated is to pause the NPWT and remove the suction tubing. If the foam remains collapsed; it is a sign that a dressing change needs to be performed.

There is a special group of patients that will not benefit from expedited abdominal closure; therefore, a staged or planned ventral hernia (38) approach is advised. In our experience, this group has shown increased mortality shortly after primary fascia closure; thus, caution must be exercised. Increased risk has been observed in patients requiring high doses of vasopressors, in acute heart failure, and in patients which have oligoanuria or a mean airway pressure over 30 cm H_2_O.

## Discussion

Due to its complex nature, severe abdominal sepsis, requires novel approaches to reduce its current mortality and morbidity rates. Currently, the most common cause of severe sepsis in the surgical intensive care unit is peritonitis (39).The use of an open OA has evolved considerably in the last two decades, in which numerous TAC methods have been proposed. The lack of consensus as to which method should be utilized has led to a varying degree of success in the application of these therapies (40). Because the variability in these methods yield such uneven results, a complication rate for one therapy can increase more than threefold with another TAC method (41). Not only has the use of different techniques yielded changing results, but also the tardy instillation of the OA has also been a key factor leading to negative outcomes. We propose that an early onset of an OA with NPWT-i, as to provide timely infection source control, should facilitate prompt primary fascial closure and lead to lower complications associated with these therapies.

It is not only important to opportunely apply the necessary treatment methods in patients with abdominal sepsis but also to guarantee that these approaches are placed in the correct manner with the adequate parameters that promote correct and satisfactory use of the NPWT devices, especially those designed for use in the abdominal cavity.

There are multiple considerations to be addressed when adequately initiating NPWT-i, such as when to perform dressing changes, the amount of fluid instilled, the length of time in which the abdominal cavity contents intermix with the solution (dwell or soak time), the size and placement of the abdominal dressing, clogging or saturation of the overlying foam, and the pressure applied to the cavity.

While the inopportune onset of treatment leads to more complications, it is also important to consider that a premature fascial closure can also be harmful; therefore, certain conditions (such as oligoanuria) must caution the surgeon as to not perform fascial closure.

This method of TAC is relatively new, and few structured studies have been performed, this limits the quality and number of publications available to researchers, and prospective blinded studies have yet to be performed. We consider that currently there is a lack of a large enough body of evidence to perform a more extensive review.

## Conclusions

Due to its complex nature, severe abdominal sepsis often requires novel approaches to help mitigate its attendant mortality and morbidity. The most common cause of severe sepsis in the surgical ICU is peritonitis (28). The use of the OA technique has evolved considerably in the last two decades, in which numerous TAC methods have been proposed. The lack of consensus has led to a varying degree of success in the application of these therapies (29), but the variability in these methods has yielded uneven results (30). The use of different forms of TAC, poor patient selection, and suboptimal use of the OA technique have been key factors leading to negative outcomes. We propose that an early onset of an OA with NPWT-i, (which, in our experience, provides a more effective and continuous inflammatory source control), should help facilitate prompt primary fascial closure, and lead to improved patient outcomes.

It is essential for the operating surgeon to understand the pathophysiology and biomechanics involved with NPWT-i, in order to properly apply this treatment method in patients with abdominal sepsis. This knowledge will help minimize complications and optimize the beneficial aspects of the NPWT devices designed for use in the abdominal cavity.

The ideal therapeutic regimen for treatment of the septic patient with an OA has yet to be determined. There are multiple aspects to this form of therapy to be considered when initiating NPWT-i, such as patient selection for OA therapy, timing of dressing changes, amount/volume and type of instilled fluid, length of time in which the abdominal cavity contents intermix with the solution (dwell or soak time), as well as the frequency and negative pressure settings required to achieve a successful patient outcome. In the two cohort studies performed by the authors, there was an almost 40% reduction in mortality between NPWT-i in comparison to the NPWT group. There was a 10% reduction in fascia closure rates and the average LOS in the SICU was cut down to almost half, therefore, the patients in the NPWT-i cohort fared better than the ones treated with traditional negative pressure therapy. Although these are not prospective, blinded, randomized studies, we consider that our results do provide a glimpse of the benefits of a particular method of TAC.

It is also important to consider when and how closure of the OA is to be conducted. Physiologically significant increases of intra-abdominal pressure that contribute to organ dysfunction (such as oliguria, respiratory failure, hypo-perfusion) may require the surgeon to abandon primary fascial closure, and to opt for other viable alternatives (biologic mesh closure, planned ventral hernia).

The successful management of the septic OA patient requires a thorough understanding and mastery of the underlying pathophysiology, the relevant anatomy, appropriate surgical techniques, and the biomechanics of the NPWT devices and their use in this setting. We have found that NPWT-i is another useful adjunct in the management of these complex patients. Further research is required in order to determine the optimal therapeutic approach in the OA septic patient. We hope that our experience will contribute in this effort.

## Funding

No funding was provided for this study.

## Provenance and peer review

Not commissioned, externally peer reviewed.

## Ethical Approval

Since no specific patient data was included, we did not solicit or obtain ethics approval for this specific paper, since we do not consider it necessary.

## sources of funding

No funding was solicited or received for this research.

## Author contribution

P.S. is the main author. He took part in the writing process, literature review, and revision and editing.

A.S. took part in the writing process, literature review, and revision and editing.

L.F. took part in the literature review, revision and editing process.

All authors agreed upon the final draft of the paper and took part in the data recollection process.

## conflicts of interest

P.S. and L.F. have previously received funding from KCI for personal services rendered to the company. A.S. has not received any funding from any party.

## Research Registration Number

reviewregistry609.

## Guarantor

Pablo Sibaja Alvarez.

## Appendix A Supplementary data

AMSTAR Pdf AMSTAR Pdf
